# Assessment of the Efficacy and Safety of Ivy Leaf (*Hedera helix*) Cough Syrup Compared with Acetylcysteine in Adults and Children with Acute Bronchitis

**DOI:** 10.1155/2020/1910656

**Published:** 2020-05-04

**Authors:** Esther Kruttschnitt, Tankred Wegener, Catherine Zahner, Silke Henzen-Bücking

**Affiliations:** ^1^Max Zeller Söhne AG, Seeblickstrasse 4, Romanshorn 8590, Switzerland; ^2^CONSULTING HMP, Brückstrasse 11, Weinheim 69469, Germany; ^3^Landarztpraxis Sevelen, Velturrietstrasse 5, Sevelen 9475, Switzerland

## Abstract

**Introduction:**

Acute bronchitis is defined as a sudden inflammation of the bronchial tubes in the lung mainly caused by viral infection. It is characterized by a persistent cough which can be productive or dry. It is the most common disease in industrialized countries, and thus herbal expectorants enjoy a high popularity in many European countries due to their favorable risk-benefit ratio.

**Objective:**

The present noninterventional study was intended to gain further data on the application of a cough syrup containing ivy leaf extract EA 575® by evaluating its efficacy and safety in children and adults with symptoms of acute bronchitis. Acetylcysteine (ACC) was chosen as comparator drug (common mono preparations). *Material and Methods*. The study was conducted at 25 medical practices throughout Switzerland as prospective, open, noninterventional study.

**Results:**

At entry visit, all clinical assessments including coughing fits, sputum, dyspnoea, rales, severity of the diseases, and coughing quality were rated with moderate intensity in both groups. At the final visit after seven days of treatment, there was a comparable improvement in both groups for all assessments except dyspnoea and number of cough attacks which showed a higher improvement in the EA 575® group compared with ACC. Further, cough-associated sleeping disorders improved more in the EA 575® group. Both, physicians and patients described the efficacy of EA 575® comparable with acetylcysteine. Observations of the tolerability were comparable for both products. The study results indicate that ivy leaf extract might be an effective alternative to acetylcysteine with respect to the improvement of respiratory function in children and adults at a slightly better evaluation of efficacy.

## 1. Introduction

Acute bronchitis is a clinical term implying a self-limited inflammation of the large airways of the lung that is characterized by cough without pneumonia. Other characteristic symptoms include sputum production, shortness of breath, and wheezing related to the obstruction of the inflamed airways. Further symptoms are fever and sore throat [1]. In addition, approximately 50% of patients with acute bronchitis report the production of purulent sputum [2]. Acute bronchitis affects approximately 5% of adults annually, with a higher incidence observed during winter and fall than in summer and spring [3, 4]. Acute cough is mainly caused by viral infection [5–8]. Cough and fever are the most common symptoms of acute bronchitis in children. In the United States, acute bronchitis is the ninth most common illness among outpatients, as reported by physicians [9].

In Europe, the prevalence for cough is 33% in the population group aged 18 to 48 years [10].

The German College of General Practitioners and Family Physicians (DEGAM) guidance describes the health-economic relevance of acute bronchitis as well [11]. Diseases with the leading symptom “cough” are economically relevant as respiratory diseases are the most common reason for working incapacity and affect the quality of patient's life [2].

Cough after acute bronchitis typically persists for 10 to 20 days. Data from a large clinical trial revealed that the median duration of cough from acute bronchitis due to all causes was 18 days [2]. Cough lasting less than 3 weeks is termed acute compared with the chronic bronchitis [11].

In principle, antitussives for the treatment of cough as a symptom of an acute bronchitis should have a good tolerability and low toxicity, especially when intended to be used in children.

*Hedera helix* preparations (ivy leaf extracts) are worldwide marketed for the treatment of different diseases of the respiratory tract system and include catarrh of the respiratory passages; symptomatic treatment of chronic inflammatory bronchial illnesses; and acute inflammations of the respiratory tract accompanied by coughing (HMPC Assessment) [1].

The clinical effects of *Hedera helix* have been investigated in a total of more than 20 [12–14] clinical studies mostly in children suffering from upper airway infections. Most of these studies have been performed as uncontrolled, open postmarketing studies, some studies in a randomized placebo-controlled design, and some in a reference-controlled design.

All of the studies involved measurements of objective and subjective parameters of efficacy (spirometric and auscultation parameters such as vital capacity, forced vital capacity, and forced expiratory volume per 1 second). In some studies, patients were requested to fill out diaries with daily assessments of, e.g., cough frequency, expectoration, and breathlessness.

Most studies were conducted with preparations containing the ivy leaf dry extract EA575® as active ingredient [12]. The same extract is contained as active ingredient in the present noninterventional study. Results of trials with different extracts are only valid for the tested preparation but not for the tested plant [15].

## 2. Materials and Methods

### 2.1. Study Medication

PROSPANEX® Cough Syrup: 5 ml contains 35 mg *Hedera helix* leaves dry extract (DER 5–7.5 : 1) as active ingredient, manufactured by Engelhard Arzneimittel GmbH and Co. KG. Recommended dosage according to the Swiss patient leaflet: for children six years and older, 5 ml, three times daily, and for adults, 7.5 ml, three times daily.

ACC mono acetylcysteine preparation (granulate, syrup, or effervescent tablet, products available in the Swiss market):

The following doses are recommended according to the summary of product characteristics. Granulate: for 12 years and older and adults, three times daily, one sachet 200 mg, and for children up to 12 years, three times daily, one sachet 100 mg. Effervescent tablet: for 12 years and older and adults, one time daily, one tablet 600 mg. Syrup: for children up to 12 years, 5 ml, three times daily, and for 12 years and older and adults, 10 ml, three times daily.

### 2.2. Design

The study was conducted as prospective, open, noninterventional cohort study by ambulant physicians (such as general practitioners, internists, paediatricians, pneumologists, and otorhinolaryngologists) throughout Switzerland.

### 2.3. Participants

Totally, 25 physicians were recruited in Switzerland who participated with 139 patients. The first patient was recruited in January 2017, and the last visit of the last patient was in March 2018. The criteria for inclusion of the patient's data in the study documentation were as follows: signing the informed consent, age six years and older, and having symptoms of an acute bronchitis accompanied with productive cough for at least 3 days for whom a therapy with EA 575® or ACC is indicated according to the physician. Criteria for exclusion of the documentation were as follows: known hypersensitivity to the active ingredients, treatment with the medications in the last two weeks before study entry, participation in another trial in the same time, and legal incapacity.

### 2.4. Ethical Consideration

The research protocol was reviewed and approved by the Independent Ethics Committee, EKOS (Ethikkommission Ostschweiz) on 16.12.2016 (BASEC Nr. 2016-01555). Before recruitment, the study procedure was explained to the subjects in detail, after which they signed consent forms. There were 4 different consent forms used in the study depending on the age of the subjects: one consent form for adult subjects, one form for subjects ranging between 14 and 18 years old (signed by the subjects), one form for subjects between ages 11 and 13 years who can already read the document by themselves (signed by the parents), and one form for parents of subjects between 6 and 11 years (signed by parents). Each patient had the right to terminate the study participation at any time.

### 2.5. Prescription Procedure

The physician selected the patients for whom the diagnosis enabled them to be included into the study and to be treated with the abovementioned prescriptions. This covered the indication of acute bronchitis and included the typical symptoms such as cough, sputum (phlegm) production, shortness of breath, and wheezing related to the obstruction of inflamed airways.

The physician was totally free in his or her decisions of therapy. It is important to note that the decision to treat the patient with either EA 575® or an ACC mono preparation was made before it was decided to include the patient into the noninterventional study.

At the inclusion visit, demographic and anamnestic data as well as the indication and typical complaints were recorded in the case report form. Subsequently, treatments were prescribed by the doctor. During the final visit, after approximately 1 week, information on the therapy and the course of the symptoms was documented.

The following clinical findings were documented during both visits by the physicians in accordance with the Bronchitis Severity Score (BSS) [16] by a 5-stage assessment: coughing fits, sputum, rales/rhonchi, and dyspnoea during coughing. Further, the severity of the disease was rated by a 7-stage-score and coughing quality (auscultation) by a 3-stage-score.

Patients themselves rated the following symptoms that were queried by the physician: 5-staged-score for coughing intensity, chest pain during coughing, dyspnoea during coughing, cough-related sleep disturbances, and possibility to cough-up mucus and 4-staged-score for number of coughing fits during daytime.

Further, the patients were asked to keep a diary during the course of treatment, which includes following symptoms: number of coughing fits during daytime, coughing intensity, chest pain during coughing, dyspnoea during coughing, possibility to cough-up mucus, cough-related sleep disturbances, frequency of wake ups at night, and general well-being. The daily self-assessment of patient's documentation for days 1–6 was done with 5-stage-scored questions except for number of coughing fits and frequency of wake ups at night which have a 4-stage-score.

### 2.6. Sample Size and Statistics

There were no specifications available for the calculation of a reasonable sample size; sample size of 300 patients deemed to be sufficient based on the previous studies and practical reasons. An interim analysis, which was performed 15 months after study start, showed that data of the collected 139 patients were sufficient for an explorative analysis with the group comparison. The study was stopped, and the physicians were informed accordingly.

The data of all patients were analysed on an intend-to-treat basis, which had used at least one dose of the medication. Drop-outs were only those patients, for which no data after intake of the medication were available.

The evaluation of the individual data on symptoms was only carried out for those patients in whom the symptom was present at the beginning of the study.

### 2.7. Measures

For both treatments, the change from the baseline bronchitis severity score (BSS) to the score at day 7 was calculated. If the standard deviations of the difference were on average not higher than 2 score points and the alpha error = 0.05, then [17] the nonparametric Mann–Whitney would demonstrate a statistical power (beta error) of more than 90% (G^*∗*^Power, Version 3.1.9, University of Düsseldorf, Germany).

Essentially, all captured data are calculated as frequency distributions and descriptive analysis (e.g., averages, medians, standard deviations, minimums, and maximums).

Demographic parameters were compared between the study groups by chi-squared test or analysis of variance, as appropriate. Effect or tolerability data was compared by repeated general linear model analysis using data from entry and final visits as intrasubject variables, treatment as between subject factor, and sex and child/adults as covariates. The level of significance was *α* = 0.05. All tests were performed using IBM SPSS for Windows software, version 25.

## 3. Results and Discussion

### 3.1. Demographics

In total, 139 data sets were analysed: 118 from the EA 575® group and 21 from the ACC group. 47 male and 71 female subjects were included into the EA 575® group, and 11 male and 10 female subjects were included into the ACC group.

The average age was 44.3 years for the EA 575® group and 49.1 years for the ACC group. Average weight was 66.1 kg in the EA 575® group and 71.7 kg in the ACC group.

Majority of the subjects was Caucasian.

With the exception of height, each of the demographic parameters was not significantly different between the study groups (Table 1).

Out of the 139 data sets, 30 children were analysed with an average height of 136.0 cm and an average weight of 32.3 kg. 13 children belonged to the class group 6–11 years with an average height of 136.0 cm and an average weight of 32.3 kg. 7 children were noted in the class group 11–13 years with an average height of 137.1 cm and an average weight of 37.0 kg. And 10 children were between 14 and 17 years old with an average height of 166.1 cm and an average weight of 63.0 kg.

### 3.2. Anamnestic Data

Viral infection-caused acute bronchitis was the most common disease at entry visit with 53.4% in the EA 575® group and 57.1% in the ACC group, followed by other acute inflammation of the respiratory diseases with 25.4% in the EA 575® group and 33.3% in the ACC group. 24.6% of the subjects from the EA 575® group suffered from acute bronchitis caused by bacterial infection and 19.0% in the ACC group (Table 2).

At entry visit, duration of symptoms was assessed as well (Table 3). Symptoms could last up to 3 days and not longer to assure the acute situation. The average duration for symptoms at entry visit was 2.341 days in the EA 575® group and 2.33 days in the ACC group. With regard to duration of symptoms, no significant differences were observed between the treatment groups.

Regarding concomitant diseases, 44.1% of the subjects in the EA 575® group suffered from concomitant diseases and 38.1% in the ACC group. Concomitant diseases were mainly treated with antibiotics and other treatment (Table 4).

### 3.3. Safety

Of the 118 patients included in the evaluation with EA 575®, treatment was carried out over an average period of 7.48 days. This results in 118 × 7.48 = 883 patient days. In the ACC group, 21 × 7.48 = 157 patient days were calculated together across all dosage forms.

During the study, two unwanted events occurred. One patient reported at the final visit to have “discontinued treatment” after 8 days and switched to ACC due to lack of efficacy. One patient reported diarrhea after she had used EA 575® for two days, and after discontinuing of the medication, the patient had recovered. Since data were incomplete for this patient, it was not considered in the overall study assessment.

Physicians judged the tolerability as “very good” and “good” with 99.1% for the EA 575® group and 95.2% for the ACC group.

Patients or their parents rated the tolerability as “very good” and “good” with 98.3% in the EA 575® group and 90.5% for the ACC group.

### 3.4. Efficacy

#### 3.4.1. Clinical Assessment by the Physicians

**Figure 1:**
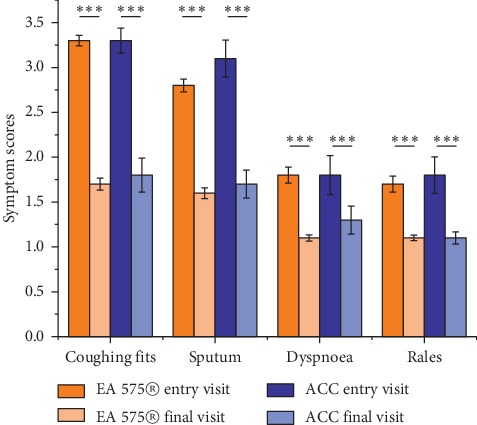
Average values of physician's evaluation comparing the final visit with the entry visit. Significant improvement in each of the symptoms from the entry visit to final visit (^*∗∗∗*^*p* < 0.001). No significant difference between the treatment groups.

The average of findings for coughing fits, sputum, dyspnoea, and rales assessed by the physicians is shown in Figure 1.

Coughing fits was the most common finding. Comparable improvement was seen for all symptoms between both groups from the entry to final visit which was statistically significant.

No statistical significance was seen between the treatments.

**Figure 2:**
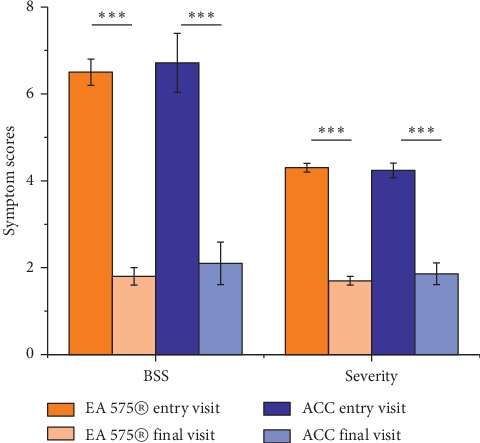
Average values of physician`s evaluation for severity of disease and calculated BSS score at entry and final visit in both groups comparing the final visit with the entry visit. Significant improvement in each of the symptoms from entry visit to final visit (^*∗∗∗*^*p* < 0.001). No significant difference between the treatment groups.

The severity of the disease and the calculated BSS showed the same result (Figure 2). There was a significant improvement in the reduction of both groups from baseline to end visit.

The severity of the disease started with 4.3 points in the EA 575® group and with 4.24 points in the ACC group showing a moderate status of disease.

At the end of the treatment, the disease was reduced in both groups to a borderline case of 1.68 points in the EA 575® group and 1.86 points in the ACC group.

At the beginning, BSS average was 6.5 points in the EA 575® group and 6.7 points in the group of ACC patients, respectively, showing a moderate impairment for both.

Over the entire treatment period, there was a reduction to 1.8 points in the EA 575® group and to 2.1 points in the ACC groups corresponding to a low impairment in both groups and showing a comparable continuous improvement in both groups.

Subgroup evaluation was performed within the EA 575® group regarding indication at inclusion into the study and concomitant disease or infection.

The results show that there is no evidence that the efficacy differs between both groups. EA 575® is effectively independent of the indication or concomitant disease.

#### 3.4.2. Clinical Assessment by the Patients or Parents

The following symptoms were assessed by the patients or parents (interview by physician):

Reduction of coughing intensity, chest pain during coughing, coughing related sleep disturbances, and possibility to cough-up mucus were comparable between both groups.

For dyspnoea during coughing, better improvement was seen in the EA 575® group.

All symptoms showed a comparable improvement in the reduction of both groups from baseline to end visit.

## 4. Discussion

Acute bronchitis is usually a self-limited condition. In most cases, only symptomatic treatment is needed [19, 20]. The value of antibiotics or other synthetic products in the treatment of otherwise healthy subjects with acute bronchitis has not been established. Further, the use of these agents is not recommended as a general practice according to present guidelines [21].

However, there are other pharmacologic treatments for impaired mucous secretion clearance including agents such as isotonic saline and acetylcysteine (also known as N-acetylcysteine (NAC)). NAC hydrolyzes the disulfide bonds of mucus proteins to decrease the mucus viscosity, thereby facilitating its clearance [22]. NAC is used as a treatment option in various conditions in which there are problems with clearance of lung mucosal secretions. A previous multicenter study showed that EA 575® exerts mucolytic action equal to acetylcysteine but with better tolerability [23]. Another noninterventional study investigated the efficacy and safety of EA 575® in school children with acute bronchitis showing the same improvement in the BSS [12].

The present Swiss study investigated the intake of EA 575® and acetylcysteine and confirmed the results from Lang et al. [12] in children and adults. The study revealed a significant and clinically relevant improvement in patients with acute bronchitis receiving oral treatment with EA 575®. This advantage could be demonstrated by the improvement of coughing fits, sputum, rales, dyspnoea, and severity of the disease during the 7 day-treatment as the primary outcome which was expressed by the BSS. Other secondary parameters from patients' assessment coming from the diary included the coughing intensity, chest pain during coughing, dyspnoea during coughing, cough-related sleep disturbances, and possibility to cough-up mucus resulting in a comparable improvement. These positive results for the herbal medication correspond to the pharmacological actions of ivy. Ivy leaves (hederae helicis folium) exert an expectorant and antispasmodic action on the respiratory tract [1]. Sieben et al. described the mode of action stating that *α*-hederin inhibited the terbutaline-stimulated internalization of the *β*2-AR. The stimulation of *β*2-AR provides an increased surfactant production which leads to the liquefaction of the mucus. Furthermore bronchial muscle cells reduce under *β*2-stimulating conditions the intracellular Ca^2+^ level. This leads to relaxation in the bronchial muscles and plausibly explains the bronchospasmolytic effect of ivy [24].

Results of this multicenter study proved the advantage of the treatment of ivy leaves in acute bronchitis that is evidently comparable with acetylcysteine. The tolerability of the herbal medication was very good. Probably the drug-related AEs (adverse drug reactions (ADRs)) were in accordance with the known side-effect profile of ivy preparations.

In the current study design (noninterventional study), the physicians were totally free in their prescription decision. Within the 139 included patients, only 21 patients were prescribed an acetylcysteine preparation resulting in an unequal group distribution.

Furthermore, concomitant diseases were treated with different antibiotics or other treatment which also might have an impact on the treatment outcome for the acute bronchitis.

This limitation can be addressed in further interventional studies with an randomized study design.

## 5. Conclusions

The current noninterventional study was performed in 118 patients using EA 575® and 21 patients using ACC for the treatment of acute bronchitis.

Clinical findings and subjective symptoms assessed by physicians showed a statistical significant improvement compared with that of the baseline, which was essentially comparable between the two groups.

In summary, the study showed that both preparations are effective and safe in the treatment of bronchitis. In addition, the EA 575® preparation showed to be effective in children and adults independent of the indication or concomitant disease and can therefore be used as an alternative to the synthetic ACC preparation.

## Figures and Tables

**Table 1:** Demographic data.

	EA 575®	ACC	Total	*P* value^*∗*^)
*Age (years)*				
*N*	118	21	139	
Adults	90; 76.3%	19; 90.5%	109; 78.4%	NS
Children	28; 23.7%	2; 9.5%	30; 21.6%	
Average (years; range)	44.3; 6–95	49.1; 9–90	45.0; 6–95	NS
*Sex (m/w)*				
*N*	118	21	139	
Male	47; 39.8%	11; 52.4%	58; 41.7%	NS
Female	71; 60.2%	10; 47.6%	81; 58.3%	
*Height (cm)*				
*N*	118	21	139	
Mean ± SEM	161.7 ± 1.8	168 ± 2.5	162.6 + 1.2	NS
*Weight (kg)*				
*N*	117	21	138	
Average (±SEM	66.1 + 1.9	71.7 ± 4.5	66.9 ± 1.7	NS
*Ethnic*				
Caucasian	103; 88.0%	16; 76.2%	119; 86.2%	NS
Asian	3; 2.6%	0; 0%	3; 2.2%	
Black	1; 0.9%	2; 9.5%	3; 2.2%	
Other	10; 8.5%	3; 14.3%	13; 9.4%	
Total	117; 100%	21; 100%	138; 100%	

^*∗*^)Analysis of variance or chi-squared test, as appropriate; NS = not significant (e.g. *p* > 0.05); *N* = number of subjects.

**Table 2:** Indication at entry visit.

Indication	EA 575®	ACC	Total	*P* value^*∗*^)
Acute bronchitis, caused by bacterial infection (ICD-10: J20.0-J20.2) [18]	29; 24.6%	4; 19.0%	33; 23.7%	NS
Acute bronchitis, caused by viral infection (ICD-10: J20.3-J20.7)	63; 53.4%	12; 57.1%	75; 54.0%	NS
Other acute inflammation of the respiratory tract accompanied with cough	30; 25.4%	7; 33.3%	37; 26.6%	NS

^*∗*^)Chi-squared test, as appropriate; NS = not significant (e.g., *p* > 0.05); *N* = number of subjects.

**Table 3:** Duration of symptoms at entry visit.

Duration of symptoms at entry visit	EA 575®	ACC	Total	*P* value^*∗*^)
The average duration of symptoms at entry visit (days)	2.41 ± 0.09	2.33 ± 0.17	2.40 ± 0.08	NS

^*∗*^)Mann–Whitney test; NS = not significant (e.g., *p* > 0.05); *N* = number of subjects.

**Table 4:** Relevant concomitant diseases and treatments.

Concomitant infections and other diseases	EA 575®	ACC	Total	*P* value^*∗*^)
No (N; %)	66; 55.9%	13; 61.9%	79; 56.8%	NS
Yes (N; %)	54; 44.1%	6; 38.1%	60; 43.2%	
Total	118; 100.0%	21; 100.0%	139; 100.0%	
*Yes, namely*				
Bronchial asthma	8; 6.8%	0; 0%	8; 5.8%	NS
Otitis media	4; 3.4%	0; 0%	4; 2.9%	NS
Rhinosinusitis	13; 11.0%	2; 9.5%	15; 10.8%	NS
Other chronic respiratory diseases	4; 3.4%	1; 4.8%	5; 3.6%	NS
Allergies	4; 3.4%	0; 0%	4; 2.9%	NS
Inflammation of upper resp. tract	21; 17.8%	3; 14.3%	24; 17.3%	NS
	EA 575®	ACC	Total	*P*-value^*∗*^)
*Treatment of concomitant diseases*				
None (N; % of patient within treatment group)	8; 6.8%	0; 0.0%	8; 5.8%	NS
Antibiotic treatment	22; 18.6%	3; 14.3%	25; 18.0%	NS
Other treatment (N; %)	23; 19.5%	8; 38.1%	31; 22.3%	NS

^∗^Chi-squared test; NS = not significant (e.g., *p* > 0.05); *N* = number of subjects.

## Data Availability

The data used to support the findings of this study are available from the corresponding author upon request.
